# *Wolbachia* Reduces the Transmission Potential of Dengue-Infected *Aedes aegypti*

**DOI:** 10.1371/journal.pntd.0003894

**Published:** 2015-06-26

**Authors:** Yixin H. Ye, Alison M. Carrasco, Francesca D. Frentiu, Stephen F. Chenoweth, Nigel W. Beebe, Andrew F. van den Hurk, Cameron P. Simmons, Scott L. O’Neill, Elizabeth A. McGraw

**Affiliations:** 1 School of Biological Sciences, Monash University, Clayton, Victoria, Australia; 2 Institute for Health and Biomedical Innovation and School of Biomedical Sciences, Queensland University of Technology, Kelvin Grove, Queensland, Australia; 3 School of Biological Sciences, The University of Queensland, St. Lucia, Queensland, Australia; 4 CSIRO Biosecurity Flagship, Ecosciences Precinct, Dutton Park, Queensland, Australia; 5 Virology, Public and Environmental Health, Forensic and Scientific Services, Department of Health, Queensland Government, Coopers Plains, Queensland, Australia; 6 Nossal Institute of Global Health, University of Melbourne, Parkville, Victoria, Australia; 7 Oxford University Clinical Research Unit, Hospital for Tropical Diseases, Ho Chi Minh City, Vietnam; 8 Centre for Tropical Medicine, University of Oxford, Churchill Hospital, Oxford, United Kingdom; The Pennsylvania State University, UNITED STATES

## Abstract

**Background:**

Dengue viruses (DENV) are the causative agents of dengue, the world’s most prevalent arthropod-borne disease with around 40% of the world’s population at risk of infection annually. *Wolbachia pipientis*, an obligate intracellular bacterium, is being developed as a biocontrol strategy against dengue because it limits replication of the virus in the mosquito. The *Wolbachia* strain *w*Mel, which has been introduced into the mosquito vector, *Aedes aegypti*, has been shown to invade and spread to near fixation in field releases. Standard measures of *Wolbachia*’s efficacy for blocking virus replication focus on the detection and quantification of virus in mosquito tissues. Examining the saliva provides a more accurate measure of transmission potential and can reveal the extrinsic incubation period (EIP), that is, the time it takes virus to arrive in the saliva following the consumption of DENV viremic blood. EIP is a key determinant of a mosquito’s ability to transmit DENVs, as the earlier the virus appears in the saliva the more opportunities the mosquito will have to infect humans on subsequent bites.

**Methodology/Principal Findings:**

We used a non-destructive assay to repeatedly quantify DENV in saliva from *w*Mel-infected and *Wolbachia*-free wild-type control mosquitoes following the consumption of a DENV-infected blood meal. We show that *w*Mel lengthens the EIP, reduces the frequency at which the virus is expectorated and decreases the dengue copy number in mosquito saliva as compared to wild-type mosquitoes. These observations can at least be partially explained by an overall reduction in saliva produced by *w*Mel mosquitoes. More generally, we found that the concentration of DENV in a blood meal is a determinant of the length of EIP, saliva virus titer and mosquito survival.

**Conclusions/Significance:**

The saliva-based traits reported here offer more disease-relevant measures of *Wolbachia*’s effects on the vector and the virus. The lengthening of EIP highlights another means, in addition to the reduction of infection frequencies and DENV titers in mosquitoes, by which *Wolbachia* should operate to reduce DENV transmission in the field.

## Author Summary

Dengue is endemic in more than 100 countries and is transmitted by the mosquito *Aedes aegypti*. The use of the symbiotic bacterium *Wolbachia* has become a potential biocontrol approach against dengue virus for two reasons. First, *Wolbachia* spreads rapidly through populations by manipulating host reproduction to its advantage. Second, *Wolbachia* limits viral replication in the mosquito by competing with the virus for essential host resources. Following field release in Cairns, Australia in 2011, the *w*Mel strain of *Wolbachia* has successfully invaded wild mosquito populations, infecting nearly all individuals. To test whether limited dengue replication in *w*Mel mosquitoes translates to a reduction in dengue transmission potential, we used a non-destructive assay to repeatedly quantify dengue virus in mosquito saliva. We found that *w*Mel significantly delayed the time it took for mosquito saliva to become infectious, reduced the frequency of dengue virus that was expectorated by mosquitoes and lowered the virus titer in mosquito saliva. We also showed that *w*Mel infection suppresses saliva production in mosquitoes that may, in part, explain our findings. The saliva-based nature of the work provides a more accurate assessment of *Wolbachia*’s ability to limit disease transmission and suggests that *Wolbachia* may have positive impacts on transmission not only by reducing the number of infectious mosquitoes in a population but also delaying the arrival of virus in the saliva.

## Introduction

Dengue fever is caused by an RNA virus belonging to the genus *Flavivirus* and is primarily vectored by the mosquito *Aedes aegypti*. Dengue viruses (DENVs) causes a spectrum of symptoms ranging from a mild fever to the life-threatening dengue shock syndrome [1–4] and collectively has become the most prevalent arthropod-borne viruses affecting humans today [5,6]. The geographic range of dengue is increasing largely due to human population growth and urbanization, especially in tropical and subtropical regions [2]. Approximately 390 million people from over 100 countries are estimated to contract dengue annually [7]. The suboptimal efficacy of a tetravalent dengue vaccine in recent phase IIb and phase III trials, and increasing insecticide resistance in mosquito populations has highlighted the urgent need to develop other alternative strategies to lessen the burden of this disease [8–10].

The use of the obligate endosymbiont *Wolbachia pipientis* has become a promising novel strategy to control dengue [9]. *Wolbachia* is a maternally inherited intracellular bacterium that is naturally found in a wide range of arthropod species including ~40% of all insect species [11]. *Wolbachia* is best known for its ability to induce diverse reproductive abnormalities in its hosts that result in its spread through invertebrate host populations [12]. *A*. *aegypti* does not carry *Wolbachia* naturally but has been stably transinfected with the bacterium [13–15]. In both semi-field cage experiments and more recently in field sites in Cairns, Australia, the *w*Mel strain of *Wolbachia* has successfully invaded natural populations of *A*. *aegypti*, risen to near-fixation within a few months of release and remained established in those sites unaided [15,16]. In laboratory studies, *Wolbachia* infection in *Aedes* and *Anopheles* mosquitoes has been shown to interfere with replication of a broad range of pathogens including viruses, filarial nematodes, bacteria and malaria parasites [17–20]. However, there are exceptions to this *Wolbachia*–mediated antiviral property. For example, contrary to most systems, *Wolbachia* infection in a *Culex* species enhanced rather than inhibited West Nile virus infection [21]. The mechanism(s) underlying *Wolbachia’s* antiviral properties in the mosquito *A*. *aegypti* are only partially understood. It has been shown that *Wolbachia* primes the innate immune system of the symbiont [17,22], competes for host resources critical for viruses [23] and manipulates the host viral defense pathways such as the microRNA pathway [24]. In laboratory-reared strains of *w*Mel infected mosquitoes captured from field release regions, this antiviral activity, as measured by reduced infectivity of mosquitoes and reduced viral titers in tissues, remained strong even one year after field deployment [25]. This evidence bodes well for the long-term stability of the *Wolbachia*-based biocontrol effect against DENV. What remains is to test the ability of *Wolbachia*-infected mosquitoes to reduce transmission of human disease in a dengue endemic region. Such trials are currently underway in Vietnam and Indonesia [9].

Vectorial capacity, that is a quantitative measure of the efficiency of a vector-borne disease’s transmission, is determined by several factors including vector density, probability of a vector biting a human, vector competence, extrinsic incubation period (EIP), and longevity [26,27]. EIP is the viral incubation period or delay between when a mosquito imbibes a dengue-infected blood meal and when the mosquito is capable of transmitting the virus to another individual. During the EIP, the virus must infect and escape the midgut, disseminate to the mosquito hemocoel and finally infect the salivary glands where it may be secreted into the saliva. EIP is a critical factor epidemiologically, as the earlier a pathogen is secreted through the saliva, the more humans the vector has the potential to infect over its lifespan [27]. This is particularly the case for *A*. *aegypti* as it tends to acquire multiple blood meals during a single gonotrophic cycle every 1–2 days [28]. Without a small animal model, the best proxy for infectiousness of the mosquito is the detection of the virus in mosquito saliva [29].

Measuring the transmission potential of mosquitoes is technically difficult due largely to the uncertainty of when a mosquito may feed and the small volume that mosquito salivate. Using a forced salivation method on pooled samples, *w*Mel-infected mosquitoes were shown to be less likely to expectorate saliva-containing DENV compared to wild-type (WT) mosquitoes [15] at 14 days post-infection (DPI). Here we use a non-destructive method to repeatedly sample pools of DENV-infected mosquitoes to assess the effect of *w*Mel on the EIP of mosquitoes recaptured from the *Wolbachia* release site in Cairns, Australia. Our work demonstrates that *w*Mel induces a delay in virus arrival in mosquito saliva. In addition, we show that *w*Mel reduces the frequency that DENV can be detected in the saliva as well as its titer. Lastly, this study reveals that higher titers of DENV in the blood meal lead to shorter EIP, higher saliva DENV titers and reduced survival of the mosquitoes.

## Methods

### Mosquitoes

Two populations of mosquitoes were used in this study. WT mosquito (not infected with *Wolbachia*) eggs routinely collected from ovitraps outside the *Wolbachia* release zone in Cairns, Australia. *w*Mel-infected (*w*Mel.F) mosquito eggs were collected from *Wolbachia* release zone in mid 2012. *A*. *aegypti* species identification was based on specific morphological characteristics. All mosquitoes were screened for *Wolbachia* infection using qPCR [16]. To retain genetic diversity all WT mosquitoes were used within 4 generations of the field. To prevent drift between the two lines and maintain genetic diversity, 20% of the males in the *w*Mel.F line were replaced each generation with WT males. After hatching, larvae were reared at a standard density of 150 individuals per 3 L of distilled water in 30 x 40 x 8cm plastic trays and fed fish food (Tetramin Tropical Tablets, Tetra, Melle, Germany) until pupation. Pupae were transferred to 30 x 30 x 30cm cages to allow adult emergence at a density of approximately 400 individuals per cage. All mosquitoes were maintained in a controlled environment insectary at 25°C, ~70% relative humidity, with 12:12h light:dark cycle. Adults were allowed to feed on a 10% sucrose diet *ad libitum*.

### Viruses

A dengue virus serotype 3 (DENV-3) strain, which was originally isolated from a patient during the 2008/2009 outbreak in Cairns [30] was used in this study. It had been passaged five times in *Aedes albopictus* C6/36 cells to generate sufficiently high titer for infection. Virus was propagated, harvested and stored in single-use aliquots as previously described [31]. Virus stocks were titrated using plaque assays to a titer of 2 x 10^7^ plaque forming units (PFU)/ml. This strain was selected for this study because it is capable of achieving a high titer to maximize the chance of infecting the mosquitoes following ingestion. The strain was also responsible for causing one of the largest outbreaks in recent history (>900 cases) in far north Queensland. Only 5% of circulating strains in the region have led to more than 100 cases of disease in a single outbreak with the next largest after the 2008/2009 outbreak causing >400 cases in 1997/1998 [30].

### DENV infection

For infection, five to eight day old female *A*. *aegypti* were deprived of sucrose for approximately 18 h and then allowed to feed on a blood meal consisting of defibrinated sheep blood mixed with an equal volume of 2 x 10^7^ PFU/ml to a final DENV concentration of 1 x 10^7^ PFU/ml through a piece of desalted porcine intestine stretched over a water-jacketed membrane feeding apparatus for three hours at 37°C. Blood-engorged mosquitoes were sorted the following day under CO_2_ and placed in 250mL cups (Sarstedt, Germany) in groups of ten. The entire experiment was repeated with 1 x 10^6^ PFU/ml of DENV.

To ensure that virus in sheep blood remains infectious over the duration of the feed, aliquots of the mixture of DENV-3 with sheep blood were taken at zero and four hours in a separate experiment also carried out at 37°C. Live infectious virus was serially diluted and titered in the wells of a 96-well microtiter plate seeded with confluent monolayers of C6/36 cells. Plates were incubated for 10 days and fixed with PBS/acetone. Virus infection was identified in the fixed cell monolayers using a cell culture enzyme immunoassay [32]. The flavivirus-reactive monoclonal antibody, 4G2 (TropBio, Townsville, Australia), was used as the primary antibody [30]. The titer as measure in tissue culture infective dose (TCID_50_) of the DENV-3 remains stable over a four-hour period (S1 Table).

### Saliva collection

Saliva was collected from mosquitoes in groups of ten using a method that exploits the fact that mosquitoes expectorate virus when they sugar feed [33]. Blood-engorged mosquitoes were briefly anaesthetized under CO_2_ and placed in each 44mm x 55mm (diameter x length) 250ml polypropylene cups (Sarstedt, Germany) covered by a piece of 100% polyester curtain lace (Spotlight Pty Ltd, Australia). Saliva was collected in 10.8mm x 46mm (diameter x length) 2ml polypropylene screw-cap tubes (Sarstedt, Germany). The cap of a 2ml polypropylene screw-cap tube was attached to the bottom of the inside of the 250ml cup containing the mosquito using a small piece of adhesive plasticine (Bostik, Thomastown, Vic, Australia). Two hundred μl of 10% sucrose solution was then pipetted into the cap; this was the only source of food and fluid for the mosquito. It was expected that mosquitoes would expectorate into the sucrose during feeding. Mosquito survival was recorded before each collection. During the collection, the mosquito was anaesthetized under CO_2_ and the pre-labelled body of the 2ml tube was carefully screwed onto the cap containing the 10% sucrose solution. The sealed tube was then removed from the cup, a new cap was affixed to the bottom of the 250ml cup, and new sucrose solution was pipetted into the cap. This method eliminates the requirement to pipette small volumes of expectorate and sucrose solution, which would likely to result in a loss of material. Tubes were then stored at -80°C. Collections were made every day from four to 14 days post-infection (DPI) and all samples were visually screened for mosquito body parts and blood spots from regurgitation or feces at collection. None were found.

Pilot experiments were carried out to determine that DENV RNA would maintain its integrity in the 10% sucrose by spiking caps containing 200μl of 10% sucrose solutions with 10μl of serial dilutions of stock DENV, ranging from 10^7^ to 10^3^ copies of DENV. The spiked sucrose solutions were then held at 25°C, ~70% relative humidity and collected at day 0, 1, 2 and 5. DENV remained detectable at least 5 days post-inoculation with no sign of decrease in RNA copy number (S1 Fig).

### Validation of salivation assay

To determine how sucrose positive results correlated with other measures of DENV positivity we examined salivary glands and saliva via forced feeding. First DENV fed mosquitoes were provided food dye (Queen fine food Pty. Ltd. Australia) containing 10% sucrose solution in the standard cap assay four days after blood feeding. After a further 24 hours mosquitoes were visually inspected for food dye in their crops. Those that had fed on the sugar solution were then forced to salivate into a capillary tube [34,35] containing sterile RPMI1640 medium (Invitrogen). This medium was used instead of the standard mineral oil because the oil was found to interfere with the efficacy of the downstream RNA extraction. With this modification salivation could not be confirmed visually by the formation of bubbles as is possible with oil. The midgut, head and salivary gland were dissected from the individual after salivation and prepared for DENV quantification. As a negative control, a tube cap containing sucrose was maintained at the bottom of the cup. The body of the tube was tightly screwed to the cap to prevent infected mosquitoes from salivating into the sucrose. All were negative for DENV.

To determine the likelihood of occurrence of false positive in the sucrose feeding salivation assay due to contamination of mosquito fecal material, we carried out saliva collections with a modification to the mosquito housing. A false floor made of mesh (opening 1.44 mm^2^) was laid over the bottom of the container preventing direct contact from the mosquito and the sucrose collection cap but allowing fecal matter to pass through. Mosquitoes were orally infected with live virus (4 X 10^6^ genomic copies/mL) and were given sucrose on the top of the housings covered in mesh using cotton wools. A total of 45 WT and 50 *w*Mel.F dengue fed mosquitoes were held in these containers from 1 to 15 DPI. Sucrose cups were collected and checked for presence of DENV every two days. All housings were scored for visual evidence of fecal contamination below the mesh and sucrose caps that contained visible blood excluded from further analysis.

### DENV extraction

Samples were thawed at room temperature and 200μl of Lysis Buffer containing 5.6 μg of Carrier RNA from a PureLink Pro 96 Viral RNA/DNA Kit (Life Technologies, Carlsbad, CA, USA) was added to each tube. Approximately 80μl of the sucrose solution evaporated during a day period between collections, resulting in an increase in viscosity of the remaining solution. We ensured that the sucrose solution was mixed well with the Lysis Buffer containing the Carrier RNA by shaking the tubes in a mini Bead Beater (Biospec products, Bartlesville, Ok, USA) for 1 min. The use of the Carrier RNA was essential as it both binds to viral RNA to increase its affinity for the silica matrix and reduces any viral RNA degradation from nucleases that may be present in the sample. Viral RNA was then extracted using the PureLink Pro 96 Viral RNA/DNA Kit according to manufacturer’s instructions. The EIP for each replicate was defined as the first time point at which DENV became detectable in saliva.

### DENV quantification

A two-step approach was used to synthesize cDNA of DENV RNA and subsequent quantification using qPCR as previously published [31]. Viral titer was expressed as dengue virus copy number per part using absolute quantification. A 107-bp fragment from the 3’ UTR region of the DENV (that is, in the same region that the primers amplify) was amplified and cloned into the pGEM-T vector system (Promega, Madison, WI). The plasmid was transformed into *Escherichia coli*, extracted using phenol-chloroform, and linearized by restriction enzyme digest. The copy number of the linearized plasmid was measured using the NanoDrop spectrophotometer. A standard curve of 10^6^, 10^5^, 10^4^, 10^3^, 10^2^, 50 and 10 of DENV fragment copies was constructed from a serial dilution. The limit of detection was set at 10 copies for this study, as it is the last dilution of the standard curve that amplified at least 95% of the time in 28 replicates. The concentrations of DENV in the samples were extrapolated from the standard curve and expressed as concentration per part by back calculating to the initial concentration of RNA [36].

### Mosquito saliva volume measurement

Saliva volume was measured at five different ages (5, 11, 17, 23 and 30 days) spanning the lifetime of the mosquitoes to assess whether failure to detect DENV may be the result of low saliva production. Mosquitoes were starved for approximately 18 hours prior to undergoing forced salivation into mineral oil. The diameter of the saliva droplets was measured using an ocular micrometer at 40 X magnification. The volumes of the droplets were calculated using the sphere formula as previously published [34,37].

### Mosquito feeding frequency

To determine if *Wolbachia* infection and/or the age of the mosquitoes affect feeding frequency, feeding rates were monitored in WT and *w*Mel.F mosquitoes (~35 individuals each) post DENV feed (as above) using food dye colored sucrose. At 5,11 17 and 24 DPI mosquitoes were randomly allocated 10% sucrose dyed either with 5% blue or pink food coloring (Queen fine food Pty. Ltd. Australia). After 24 hours, mosquitoes were visualized under a dissecting microscope and then given the alternate colored sucrose solution. Visual inspections were then carried out every 24 hours. Once an individual mosquito was scored for the number of days required for the second dye to arrive in the abdomen it was removed from the assay.

### Data analysis

EIP was analyzed using a general linear model with an identity link function and normally distributed errors with *Wolbachia* infection status, DENV titer of bloodmeal and their interactions as factors. Saliva volume and feeding frequency data were analyzed using a general linear model with an identity link function and normally distributed errors. Mosquito age and *Wolbachia* infection status were tested as factors. The number of days infective and saliva DENV titer were analyzed using Mann-Whitney *U* tests due to deviation from normality. Saliva DENV titer was analyzed using a generalized linear model with a logit link function and a normal error distribution. Survival data were analyzed using Kaplan-Meier analysis and log-rank statistics. Statistical analysis was performed with the software Statistica 8.0 (Statsoft, Inc. USA).

## Results

### Validation of saliva assay

There was very high agreement and significant correlation between sucrose feeding salivation assay positive for DENV and both DENV infection of the salivary gland and forced salivation results (Table 1). In only one case was a sucrose feeding salivation positive result not recapitulated by the DENV infection in the salivary gland. The midgut and head tissues from this same mosquito were positive, however, which may indicate a possible failure of the RNA extraction for the salivary gland tissue. The sucrose feeding assay identified a greater number of positive samples than forced salivation (10 vs 6). In three out of four the cases the salivary gland, head and midgut of these mosquitoes were also DENV positive. These differences are not surprising given that the forced feeding assay is a poor baseline control for salivation for several reasons. First, without visual confirmation of salivation due to the requirement of using medium for collection purposes not all of the mosquitoes will have participated in forced salivation. From our saliva volume assay with forced salivation in mineral oil (n = 547) we estimated that mosquitoes fail to expectorate 13.2% of the time. Second, forced expectoration can produce incredibly small volumes, from 0.11 to 23.63 nl. Despite flushing the capillary tubes with the medium the entire contents is cannot be completely collected. Third, because the mosquitoes were forced to expectorate after the 24 hr sucrose collection window there may be real differences in whether they are secreting virus and hence positivity of samples.

**Table 1:** Summary data and Spearman’s correlation for sucrose feeding salivation validation assay.

Comparison	# DENV positive out of 47 fed	R_s_	t	df	P
sucrose feeding salivation assay vs salivary gland infection	10 vs 9	0.87	11.77	45	P < 0.0001
sucrose feeding salivation assay vs forced expectoration assay	10 vs 6	0.73	7.19	45	P < 0.0001

The design of the fecal contamination assay was effective as feces were present below the mesh in 100% of the housings. The rate of DENV positivity in sucrose due to strict fecal contamination ranged from 0% to 4.8% but averaged 0.88 and 0.25% for WT and *w*Mel, respectively, across all time points (S2 Fig).

### EIP

**Fig 1:**
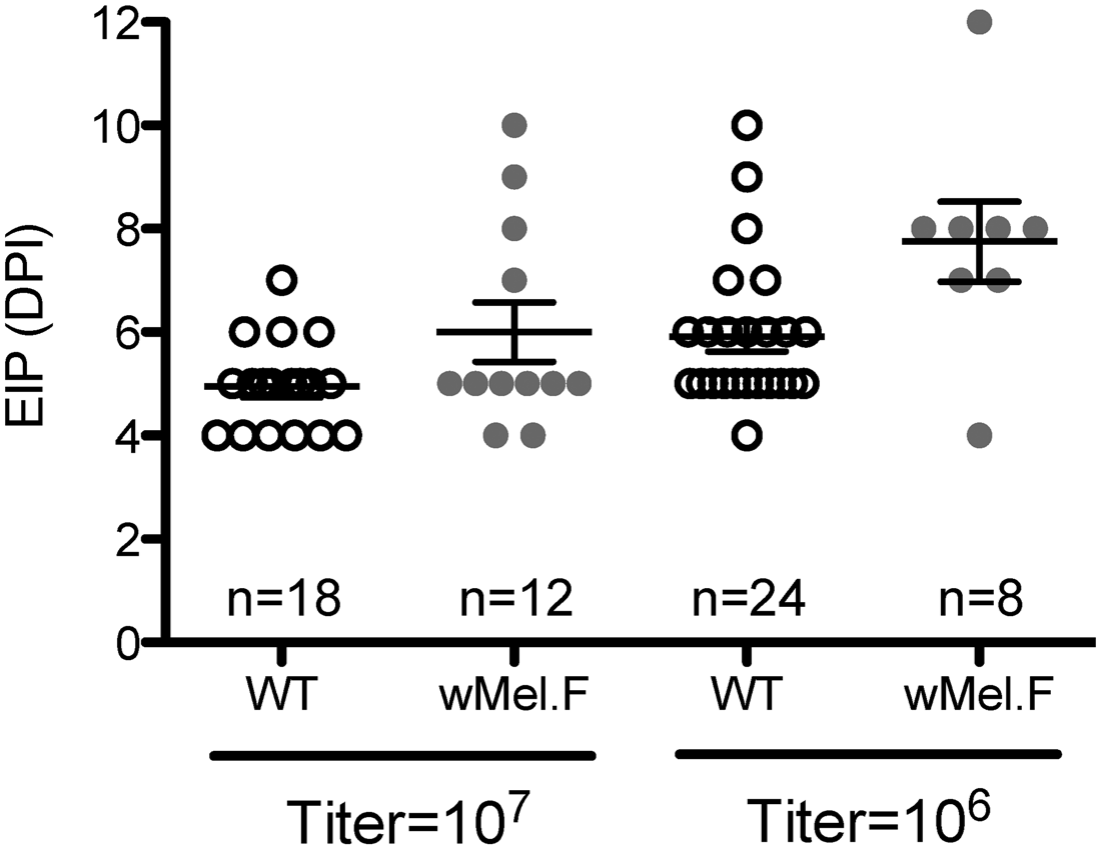
Extrinsic incubation period (DPI) of WT (white) and *Wolbachia* infected (*w*Mel.F) mosquitoes (grey). Bars depict means ± S.E.M. Mosquitoes were orally infected with either 10^7^ or 10^6^ PFU/ml of DENV. Each data point represents a pool of 10 mosquitoes.

Two different titers of DENV blood meal were used to orally infect the mosquitoes: a high titer blood meal of 10^7^ PFU/ml and a lower concentration of 10^6^ PFU/ml. The EIP for each replicate was defined as the first time point at which DENV became detectable in the sucrose feeding solution. Overall there was an significant main effect of *Wolbachia* infection (Fig 1) with *w*Mel.F experiencing a lengthier EIP compared to *Wolbachia*-free WT mosquitoes (df = 1, *F* = 11.6, *P*<0.01). There was also a significant difference in EIP due to blood meal titer (df = 1, *F* = 10.3, *P*<0.01). There was no significant *Wolbachia* by blood meal titer interaction effect (df = 1, *F* = 0.84, *P* = 0.36) on EIP.

When mosquitoes were infected with the high titer blood meal, the mean EIP of *w*Mel.F was 6.0 ± 0.58 DPI as compared to 4.9 ± 0.21 DPI in WT mosquitoes. When mosquitoes were infected with a 10^6^ PFU/ml DENV blood meal, the mean EIP of *w*Mel.F was 7.8 ± 0.77 DPI compared to 5.9 ± 0.29 DPI in WT mosquitoes (Fig 1). This difference suggests that the *w*Mel slows the arrival of virus in the saliva, particularly at a lower orally infected virus concentration.

### Number of days infective

**Fig 2:**
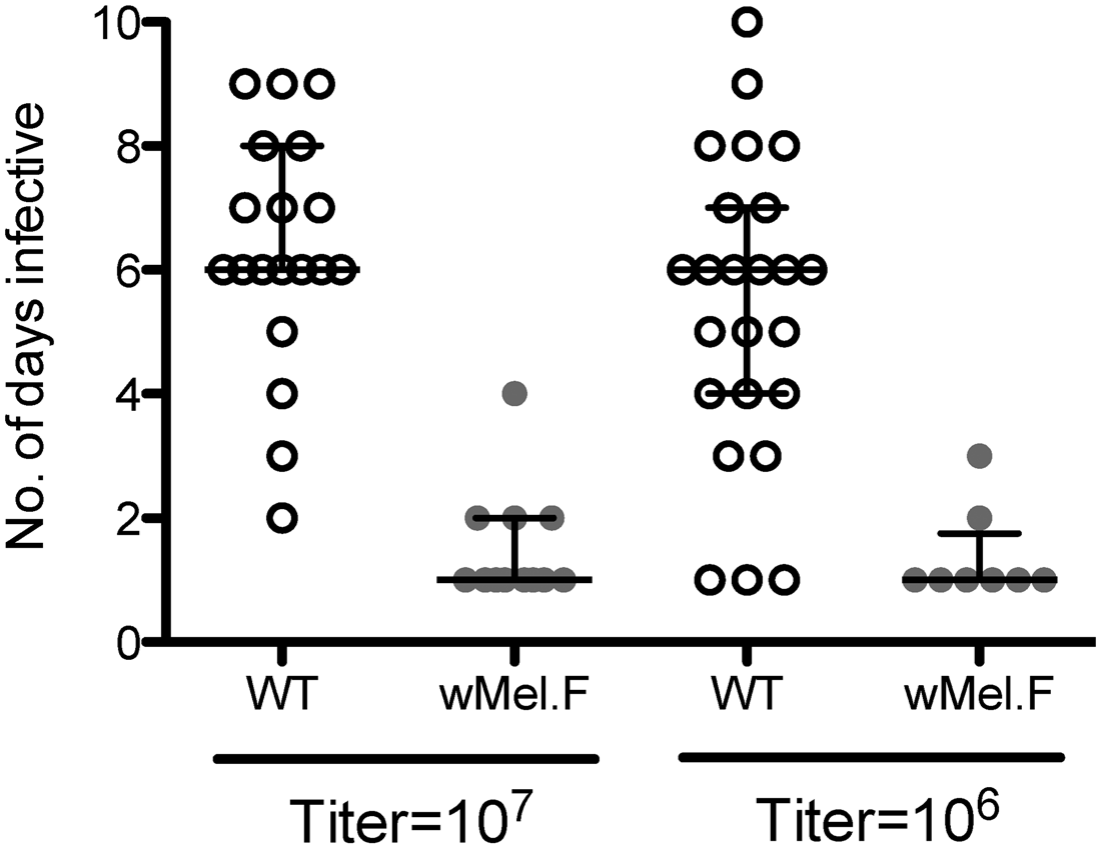
Number of days infective saliva was detected for WT (white) and *w*Mel.F mosquitoes (grey). Bars depict medians ± interquartile range. Mosquitoes were orally infected with either 10^7^ or 10^6^ PFU/ml of DENV. Each data point represents a pool of 10 mosquitoes.

A biological replicate (a pool of mosquitoes in a cup) was considered infective for a particular time point if DENV was detected in the sucrose solution on that day. Overall, *w*Mel.F exhibited fewer infective days compared to WT mosquitoes (Fig 2). When mosquitoes were infected with a 10^7^ PFU/ml DENV blood meal, the presence of *w*Mel reduced the median number of days infective from 6 days to 1 day (*Z* = 4.47, *P*<0.0001). The same differential was seen when mosquitoes were infected with 10^6^ PFU/ml DENV blood meal (*Z* = 3.48, *P*<0.001).

### DENV titer in saliva

**Fig 3:**
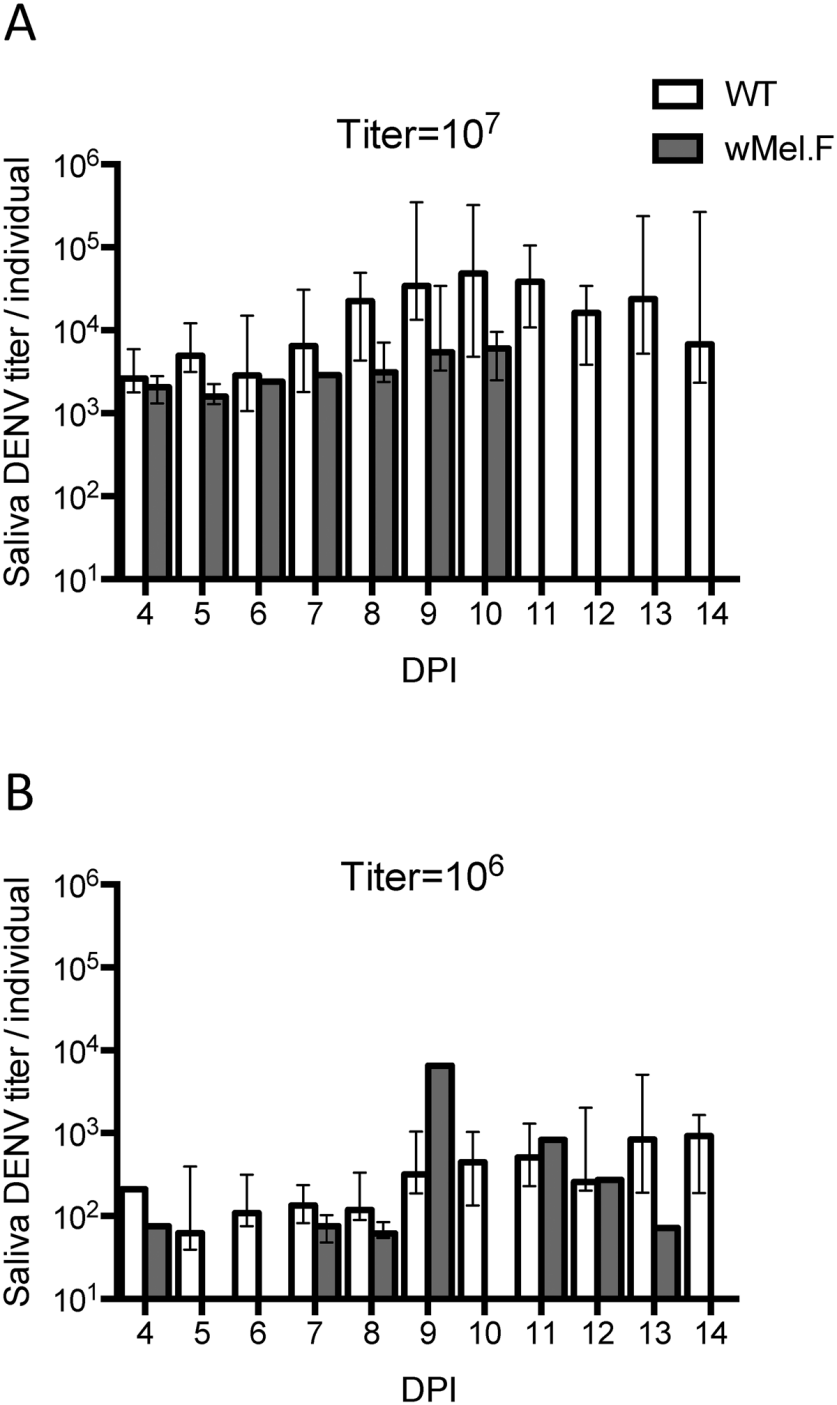
The DENV titer in the sucrose solution of WT (white bar) and *w*Mel.F mosquitoes (grey) fed on 10^7^ (A) or 10^6^ (B) PFU/ml of DENV. Bars depict medians ± interquartile range. DENV titer is expressed as copies per live individual to correct for death.

The presence of *w*Mel also reduced the amount of DENV in the mosquito saliva. After a high DENV titer blood meal (Fig 3A), the median copy number of DENV in *w*Mel.F mosquito saliva was 2633 copies across all timepoints measured, a 4.9 fold decrease as compared to the median copy number of 12826 in WT mosquitoes (*Z* = 3.51, *P*<0.001). It is also noted that no DENV was detected in saliva from *w*Mel mosquitoes after 11 DPI. This is likely a stochastic result due to low infection frequencies (unique to *w*Mel) and declining population sizes due to age associated mortality.

When mosquitoes were infected with a low titer blood meal (Fig 3B), the presence of *w*Mel lowered (2.6 fold) the median copy number of DENV in mosquito saliva from 198 copies to 76 copies (*Z* = 2.08, *P*<0.05). The amount of virus in the mosquitoes’ saliva was positively correlated with the titer of DENV used to infect the mosquitoes. WT mosquitoes expectorated more DENV when infected with a high DENV titer blood meal as compared to a low titer (*Z* = 12.3, *P*<0.0001). The same effect of DENV titer in the blood meal on saliva DENV titer was observed in *w*Mel.F mosquitoes (*Z* = 3.78, *P*<0.001). The DENV copy number varied hugely by almost 4 logs from 507 to 2458333 copies in WT mosquitoes fed with a 10^7^ PFU/ml DENV blood meal. While there are trends in titer across DPI overall the effect of day was not significant (df = 1, *F* = 0.95, *P* = 0.33).

### Mosquito survival post dengue infection

**Fig 4:**
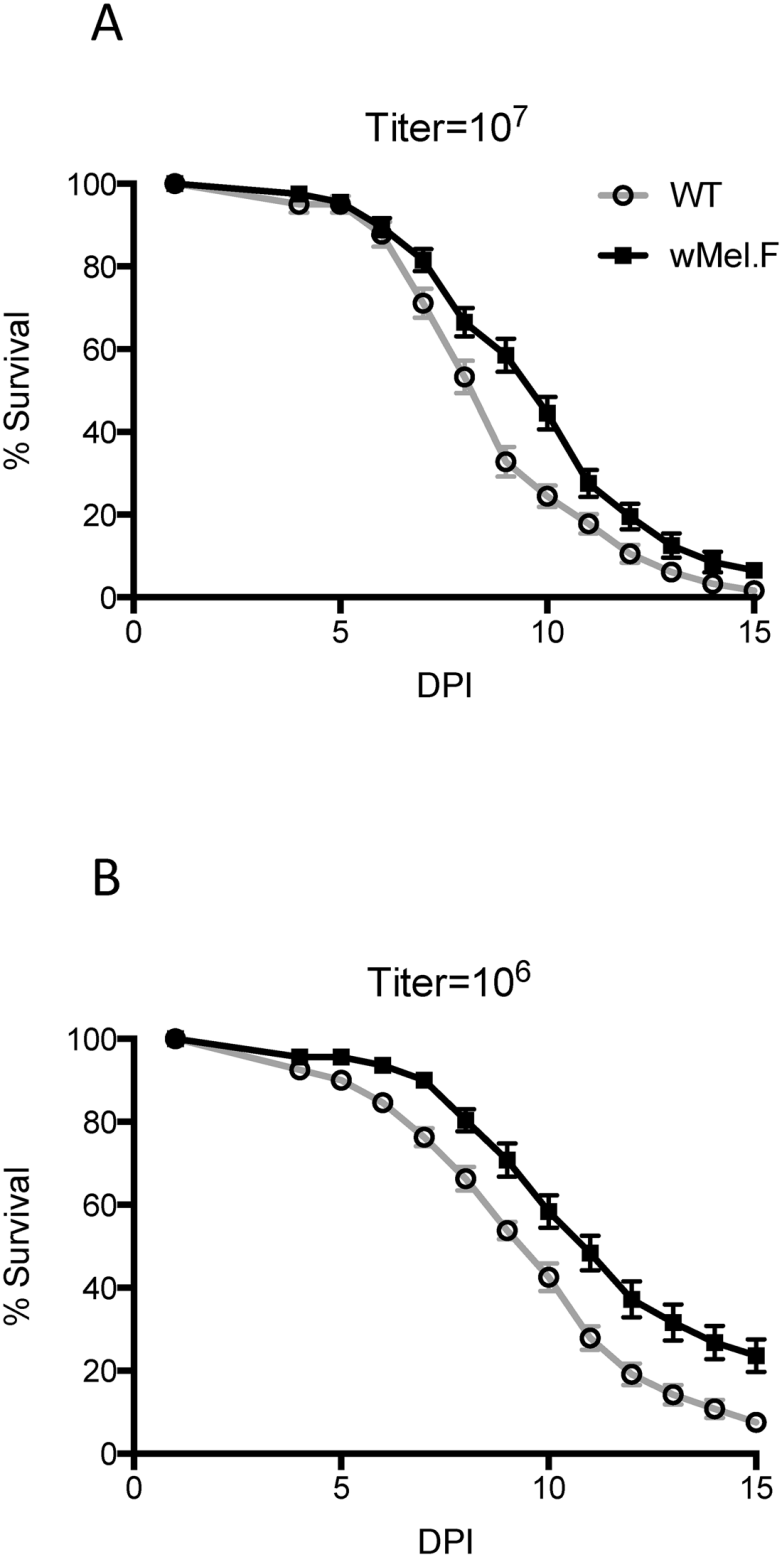
Survival curves of WT (grey line) and *w*Mel.F mosquitoes (black line) orally infected with either 10^7^ (A) or 10^6^ (B) PFU/ml of DENV. Bars depict means ± S.E.M.

The presence of *w*Mel infection lengthened the lifespan of mosquitoes as compared to WT following DENV infection. This effect was significant for mosquitoes fed both high (*P*<0.001, Fig 4A) and low (*P*<0.0001, Fig 4B) DENV titer blood meals. The titer of DENV in the blood meal was inversely correlated with mosquito survival. WT mosquitoes infected with 10^7^ PFU/ml of DENV died more quickly than those infected with the lower titer of 10^6^ PFU/ml (*P*<0.001). This suggests that DENV infection is costly to mosquitoes and that *Wolbachia* is providing some protection to the host.

### Dynamics of infective mosquitoes

**Fig 5:**
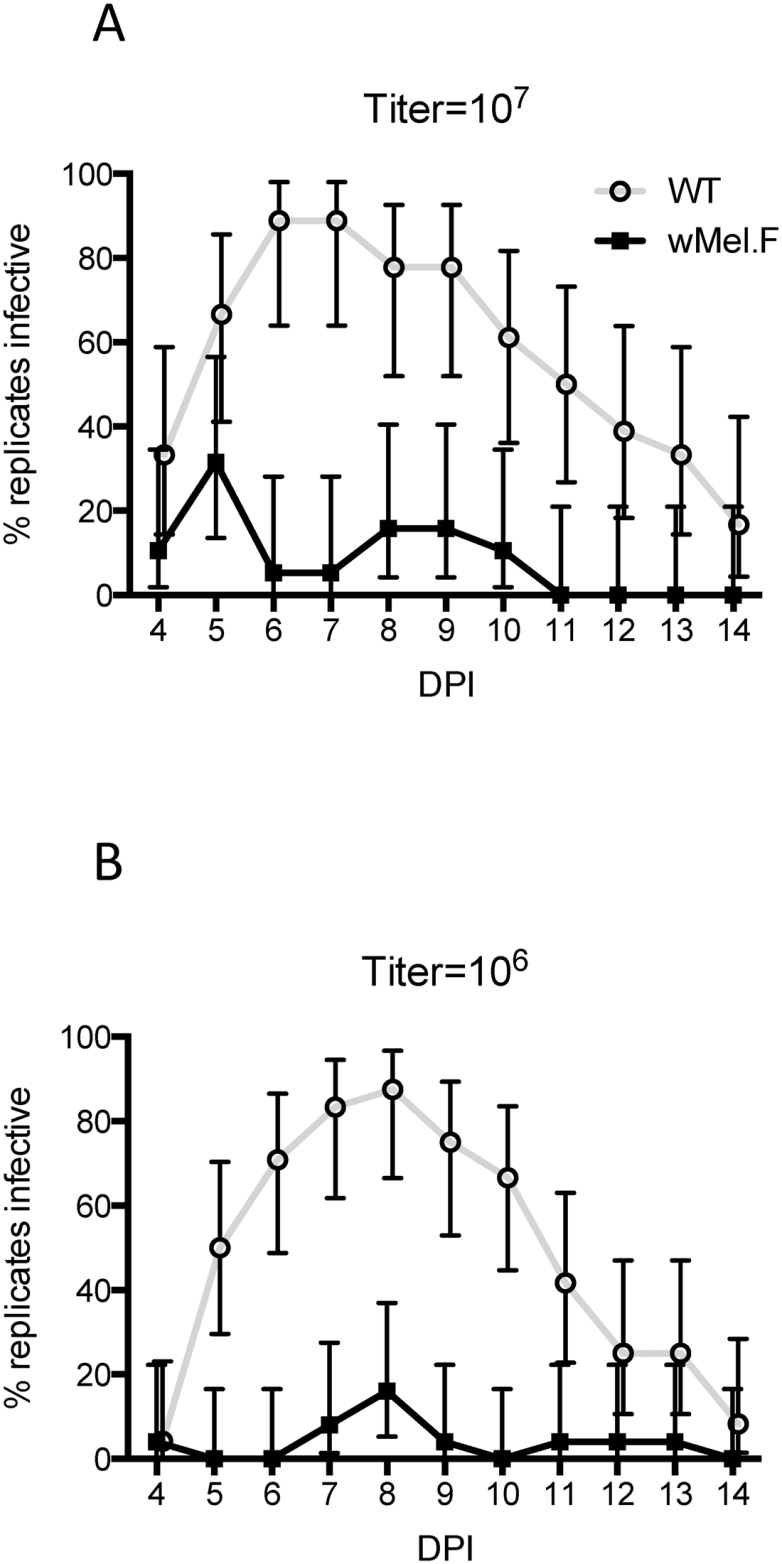
Percentage of replicates infective through time ± confidence intervals for WT (grey line) and *w*Mel.F mosquitoes (black line) orally infected with either 10^7^ (A) or 10^6^ (B) PFU/ml of DENV.

Using each cup of 10 mosquitoes as a biological replicate, we assessed the dynamics of infection of the mosquitoes over time. After a high DENV titer blood meal (Fig 5A), WT mosquitoes quickly became infective and peaked at 88.9% infective (16/18) at 6 DPI. In contrast, *w*Mel.F mosquitoes remained largely non-infective with a maximum of 31.6% (6/19) of the replicates expectorating detectable DENV at 5 DPI. A similar trend was seen when mosquitoes were fed a low titer blood meal (Fig 5B). The proportion of replicates infective declines as the mosquito age even when most of the replicates are infective at earlier timepoints.

### Saliva volume

**Fig 6:**
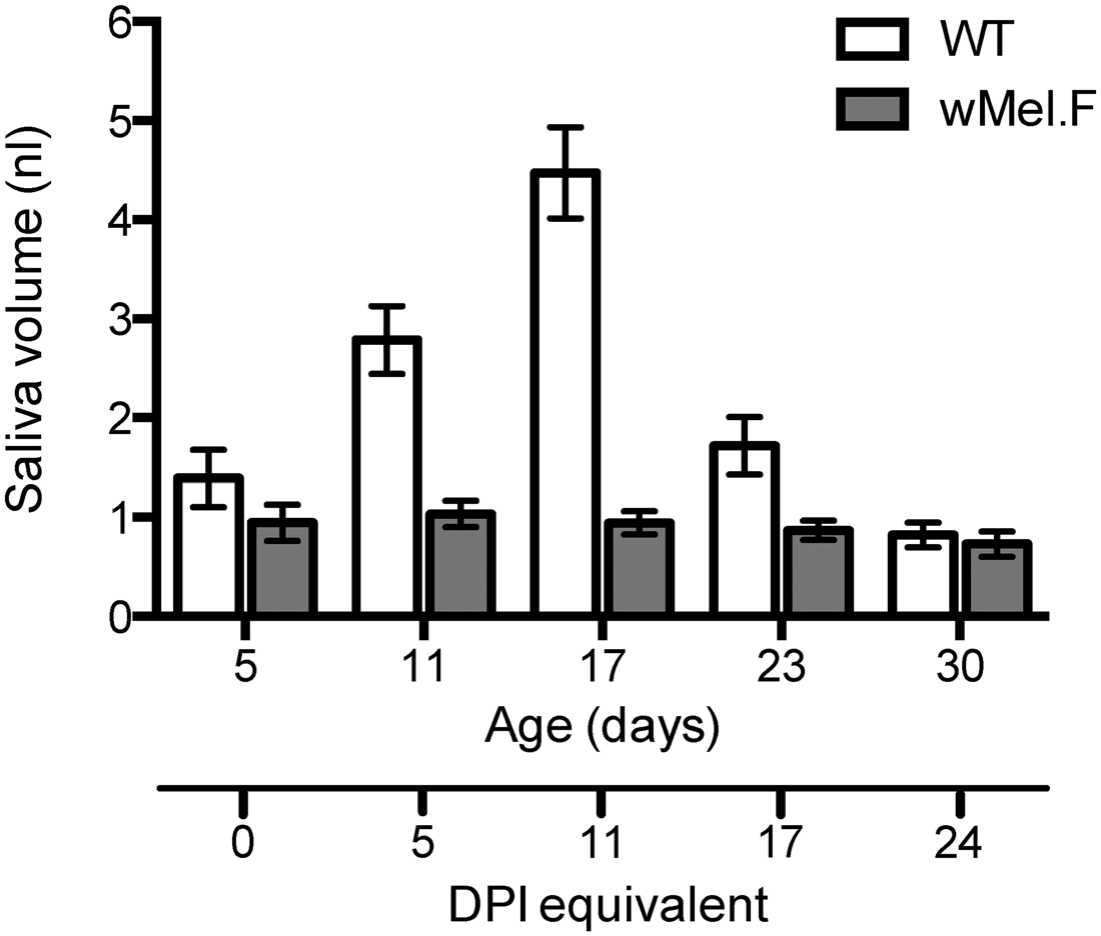
Comparison of saliva volume of WT (white) and *w*Mel.F mosquitoes (grey) of different ages. Saliva volume is measure through the sphere volume of saliva droplets using mineral oil. Bars depict means ±S.E.M. Number of replicates range from 33 to 80 with a mean of 62 individuals for WT mosquitoes and 34 to 85 with a mean of 64 for *w*Mel.F mosquitoes. 86.8% of all the mosquitoes produced saliva during the assay.

To determine if the dynamics in saliva DENV titer and infectivity can be explained by mosquito saliva production we compared the saliva volume of the two mosquito populations over time in their natural DENV uninfected state (Fig 6). *w*Mel.F mosquitoes produced less saliva as compared to WT mosquitoes (df = 1, *F* = 55.3, *P*<0.0001). The age of the mosquitoes was also a determinant of saliva volume (df = 1, *F* = 17.5, *P*<0.0001). In WT mosquitoes, saliva volume increased as mosquitoes aged until 17 days of age and then declined. In *w*Mel.F mosquitoes saliva volume remained low throughout the lifespan of the mosquitoes. The saliva volume also varied hugely between individuals especially on day 17 (ranged 0.11–23.63nL) in WT mosquitoes. The dynamics of expectorated saliva volume mirror that of DENV titer and the dynamics of the infective mosquitoes, all peaking between 5 and 11 DPI. Together, the results suggest that *w*Mel.F mosquitoes expectorate less DENV, which can at least be partly explained by producing less saliva and older mosquitoes are less likely to expectorate detectable DENV as their saliva volume deceases with age.

### Feeding frequency

**Fig 7:**
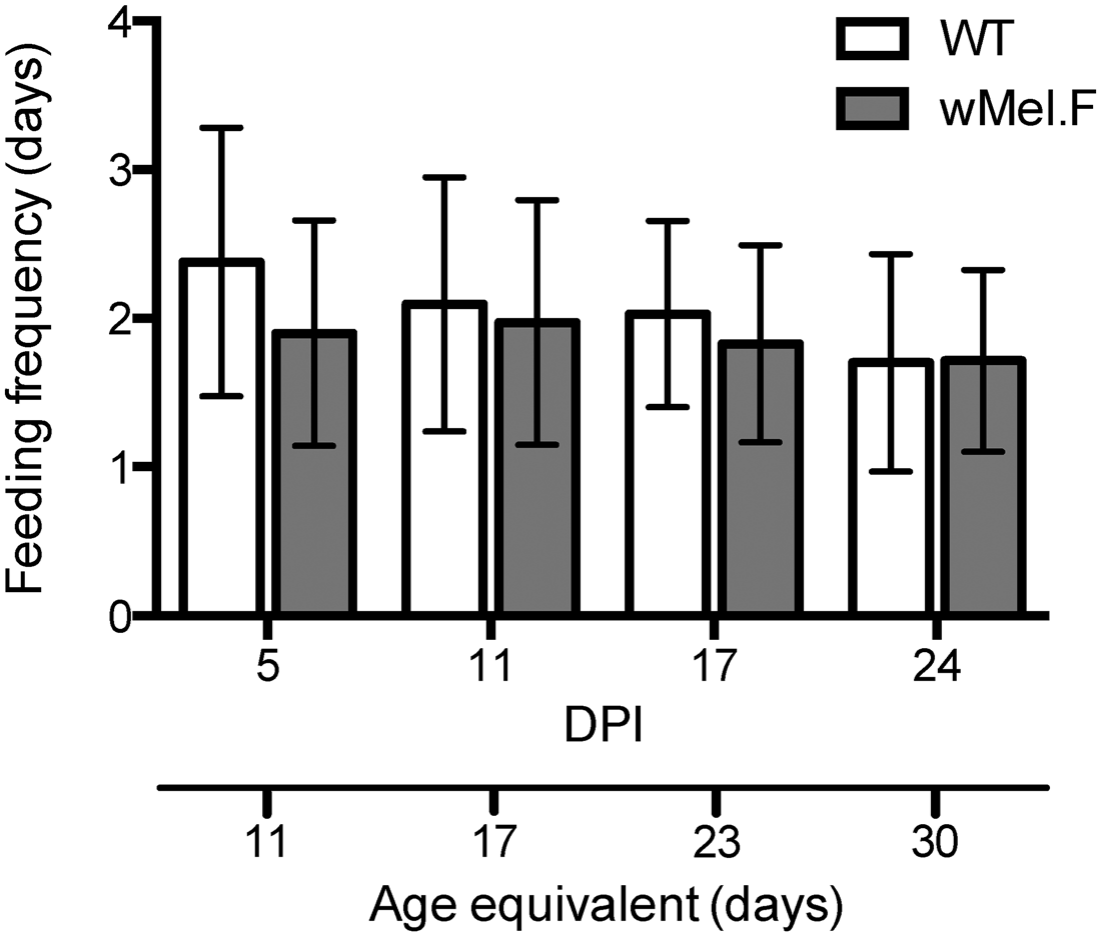
Comparison of sucrose feeding frequency of WT (white) and *w*Mel.F mosquitoes (grey) at four different ages. Bars depict means ±S.E.M. Number of replicates range from 26 to 40 with a mean of 37 individuals for WT mosquitoes and 20 to 40 with a mean of 35 for *w*Mel.F mosquitoes across all time points. 80.1% of all the mosquitoes produced feeding frequency data.

To determine if *Wolbachia* infection and/or age of the mosquitoes can affect the sucrose feeding frequency, we compared the feeding frequency of the two mosquito populations over time in their DENV infected state (Fig 7). *w*Mel.F mosquitoes fed more frequently (shorter intervals) as compared to WT mosquitoes (df = 1, *F* = 4.8, *P*<0.05). Mosquitoes also fed more often as they aged (DPI effect: df = 1, *F* = 8.0, *P*<0.01). There was no effect of food dye (df = 1, *F* = 0.074, *P* = 0.79).

## Discussion

One year after *w*Mel-infected *A*. *aegypti* field deployment in Cairns, Australia, there is no sign of attenuation in its ability to limit viral replication in the body of the mosquito and reduce viral dissemination to the head of the mosquitoes [25]. Using mosquitoes captured from the same field sites we show that the *Wolbachia*-mediated blocking effect is translatable to saliva-based measures of vector competence. Our findings show that the presence of *w*Mel not only reduces the proportion of mosquitoes with transmission potential but also delays their EIP, thus further reducing the capacity of the mosquitoes to transmit dengue. Additionally, for the first time we present data that shows that *w*Mel significantly reduces the frequency of mosquitoes that expectorate DENV and lowers virus titer in the saliva. We also offer a potential mechanism demonstrating that *w*Mel infected mosquitoes produce little saliva.

Ideally, the saliva of individual mosquitoes instead of pools would be assayed for the presence of DENV in order to better understand variation at the individual level, for infection status and saliva DENV titer. However *w*Mel reduces viral replication so effectively only a small portion of the mosquitoes display dissemination to the head tissue compared to WT mosquitoes, 6 vs 62%, respectively, as reported by previous studies with DENV-2 [15,25]. A subset of mosquitoes exhibiting dissemination may then eventually acquire transmission capability [38]. Rearing and producing infections in sufficient numbers of individuals to overcome the strength of blocking is simply intractable. This is especially the case for low-titer blood meals of 10^6^ PFU/ml as on average only ~5% of replicates were infective at any time point.

Another limitation of this study is the reliance on qPCR to detect and quantify virus. qPCR is a sensitive and efficient method to quantify DENV, however it does not differentiate infectious from non-infectious virus [36]. In contrast, plaque assays, which require much larger volumes of starting material, quantify infectious particles. While there is a strong correlation between the two measures, the estimated RNA copy number is usually 2–5 logs higher than the number of infectious units due to the presence of immature and/or defective virus [39,40]. It is therefore possible that our estimates of EIP are skewed towards earlier timepoints that could lead to an over-estimation of the mosquitoes’ transmission potential. Indeed, when using a plaque assay laboratory strains of *w*Mel mosquitoes infected with DENV-2 92-T strain were found to completely block dengue transmission as none of the pooled (0/36) mosquito saliva had detectable infectious dengue virus at 14 DPI [15]. However, the relatively poor sensitivity of the plaque assay in combination with the destructive nature of the mosquito saliva collection process made it impossible to repeatedly assay mosquito saliva from the same individual for infectious virus. It is also clear that the DENV-2 92-T strain is much less infectious in mosquitoes as compared to DENV-3 isolate used in this study. In WT mosquitoes an average blood feeding event produces ~60 percent of mosquitoes with disseminated infections [31] whereas in pilot studies our DENV-3 isolate achieved an infection rate near 100%. Future studies should examine the generality of DENV blocking and effects on transmission parameters for diverse viral genotypes including representatives of the other three serotypes.

Our estimate of mean EIP for this dengue strain in these mosquitoes ranges from 4–10 days, which is shorter than a previously estimated mean of 15 days (5 to 33 days at a 95% confidence) at 25°C for dengue viruses in general [41]. These findings from our sucrose collection assay are confirmed by our validation assay and other studies have argued that EIP of DENV is shorter than is commonly perceived. For example, DENV antigen was detected in the salivary gland in more than a third of mosquitoes examined as early as 4 DPI [42], and a small fraction of mosquitoes were found to have naturally leaky abdominal midguts that could facilitate rapid systemic infection [43]. Furthermore, our measure of EIP is in line with estimates of the total incubation period (EIP + Intrinsic incubation period) of 9–11 days obtained from patient records from the 2008/2009 outbreak during which this dengue isolate was collected [30].

After infection, DENV titer is highly dynamic within the insect [39]. A previous study examining the kinetics of DENV in the whole mosquito showed that midgut titer increases, peaks and eventually declines as mosquitoes age [42]. DENV dynamics in mosquito saliva is rarely studied due to its intractability. In one study using plaque assays on forced salivation on individual mosquitoes, it was found that the proportion of mosquitoes expectorating DENV decreased in older (21 days) mosquitoes as compared to young (6 days) mosquitoes despite 100% body infections. This suggests that old mosquitoes do not salivate detectable DENV [44], a finding that concurs with measures for our WT strain where infections decline in saliva after 8 DPI. The effect of *w*Mel on saliva production and feeding frequency, however, was more surprising given that the strain is considered to have few fitness effects on the host. More specifically, *w*Mel has no effect on the fecundity and egg viability of the mosquitoes but reduces the mean lifespan of the mosquitoes by approximately 10% [15]. Another strain of *Wolbachia w*MelPop, in *A*. *aegypti* radically reduces the mean lifespan by more than 40% and causes late acting blood feeding defects previously showed reductions in saliva production in mosquitoes at 26 and 35 days of age but not at 5 days [37]. Our study shows similar results with no effect at 5 days but differences beginning at 11 days. These data suggest that, even in the absence of other virulent effects, *Wolbachia* infection in mosquitoes may affect saliva production and mosquito behavior such as feeding frequency.

Using direct mosquito feeding on dengue patients, the infectious dose or the titer for each of the four DENV serotypes required to orally infect mosquitoes ranges from 6.3 to 7.5 log_10_ RNA copies/mL of patient plasma, although viremia in some patients may exceed 10 log_10_ [45]. We found that the high viral infectious dose is associated with shorter EIP and higher DENV titer in the mosquito saliva. This suggests that the transmission potential of the mosquito may be directly influenced by human host viremia levels.

We also show the ability of *w*Mel to offer protection against DENV, slowing the time to death and increasing survival. Whether this lengthening of lifespan can potentially enhance the vectorial capacity of *w*Mel mosquitoes requires further study. Future experiments should also be focused on the effect of patients with high viremia on EIP, especially titers that would not be achievable in the laboratory using cell culture methods.

The relationship between saliva volume and DENV titer has not been studied in *A*. *aegypti*. In the case of *Plasmodium falciparum*, saliva volume is positively correlated with the number of parasites in the salivary gland [46]. We argue that, in general, the dynamics of infective mosquitoes and DENV titer in saliva can at least be partly explained by the dynamics of saliva volume. The fact that older mosquitoes produce less saliva suggests that there is a window during their lifetime when infected mosquitoes may expectorate virus during a bite. This also points out possible shortfalls in formulas used to calculate vectorial capacity of mosquito-borne disease that do not take into consideration the epidemiological variation in virus expectoration with mosquito age. These findings are particularly important where the likely efficacy of *Wolbachia* in reducing DENV transmission is being modeled in advance of field trials.

Environmental factors such as temperature and larval nutritional status affect the length of EIP [47]. Recently, the diurnal temperature range (DTR), which reflects the degree of daily fluctuating temperature, was found to significantly change the outcome of infection and survival of the mosquitoes, and the EIP of DENV as compared to a constant temperature [48]. Similar fluctuating temperature at different mean baselines was also found to affect *Plasmodium* development and dissemination in *Anopheles* that demonstrated the generality of the effect of temperature on parasite transmission potential [49]. *Wolbachia* density, which is shown to be a determinant of viral blocking [50], is affected by temperature too [51]. Varying constant temperatures was found to alter the extent of parasite blocking in somatically *Wolbachia* transinfected *Anopheles* mosquitoes [52]. This raises the importance of not confining the study of vector-pathogen interactions to a constant rearing temperature of 25°C. As large-scale field releases of *Wolbachia* infected mosquitoes are currently underway in sites each with its unique baseline temperature and diurnal temperature range, it is paramount to understand how the tripartite interactions between mosquitoes, dengue virus and *Wolbachia* may change in terms of temperature regimes. Gut microbiota of the mosquitoes was known to influence the outcome of vector by pathogens [53,54]. How *Wolbachia* may impact the mosquito microbiota and thus influence the outcome of vector-borne pathogens also need to be investigated.

In conclusion, we found that the *w*Mel infection lengthens the EIP of DENV, reduces the frequency that the virus is expectorated and decreases the amount of DENV RNA copy number in saliva as compared to wild-type mosquitoes. A reduction in saliva production in *w*Mel mosquitoes can at least partially explain the above observations. These saliva-based traits offer more disease relevant measures of the symbiont’s effects on virus than using measures such as infection and dissemination. The shift in EIP, in particular, indicates an additional means by which *Wolbachia* could modulate virus transmission. The opposing effects of *w*Mel prolonging mosquito survival post DENV infection needs further investigation.

## Supporting Information

S1 Fig Virus integrity in sucrose solution. 10% sucrose solutions were spiked with serial dilutions of DENV of known copy number. Five replicates of virus spiked sucrose solution were collected at 0, 1, 2 and 5 days post-inoculation for each DENV dose inoculated.
(TIFF)

S2 Fig Percentage of replicates positive in sucrose collections due to fecal contamination through time ± 95% confidence intervals for WT (grey line) and *w*Mel.F mosquitoes (black line) orally infected with DENV. Mean contamination rates for WT and *w*Mel.F were 0.88 and 0.25, respectively.
(TIFF)

S1 Table Virus integrity in sheep blood. (DOCX)
