# Supplementary figures and images for: Wolbachia Reduces the Transmission Potential of Dengue-Infected Aedes aegypti
Source: PLoS Negl Trop Dis. 2015 Jun 26;9(6):e0003894. doi: 10.1371/journal.pntd.0003894 (PMC4482661; doi:10.1371/journal.pntd.0003894)

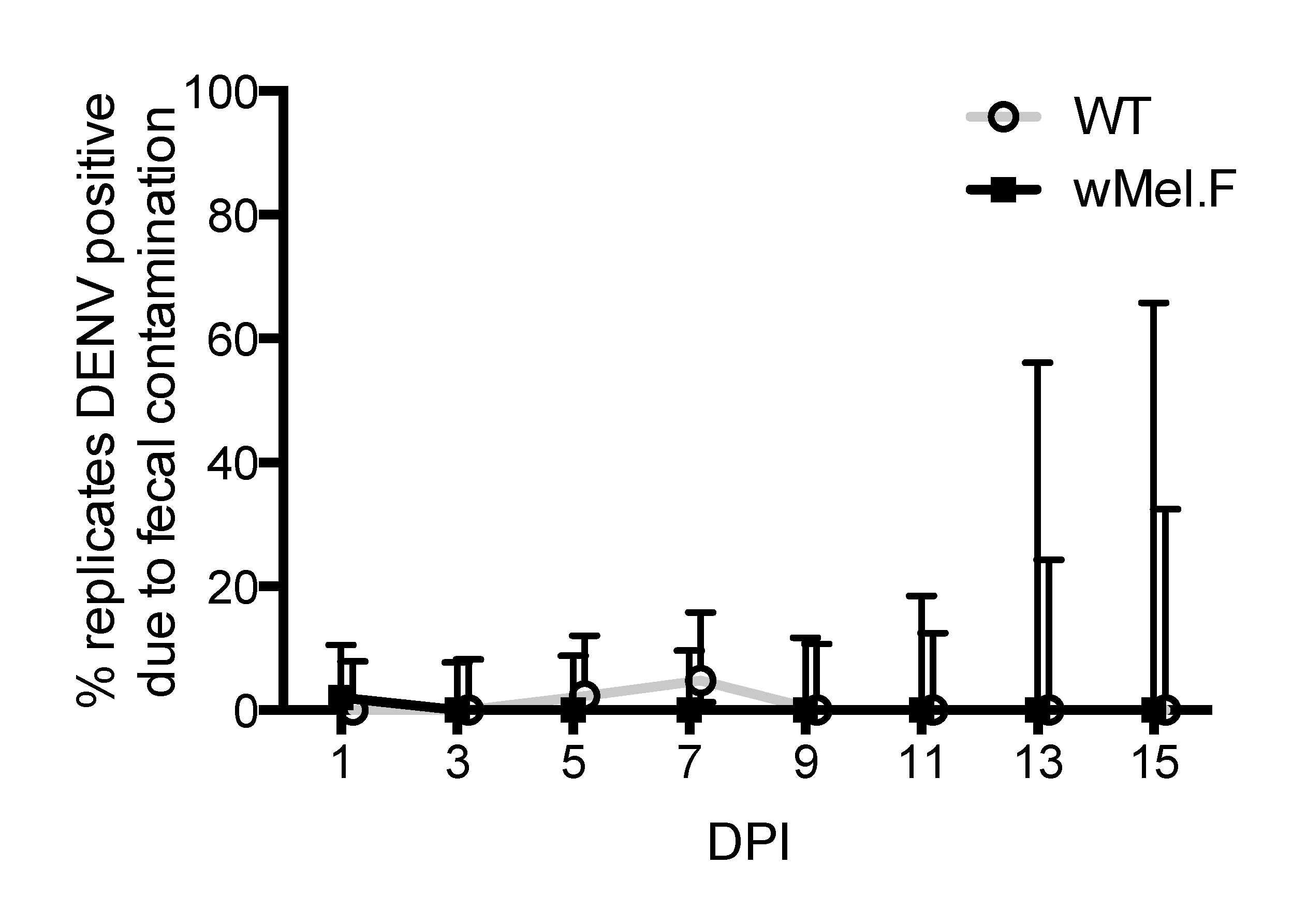

Supplement: S2 Fig — Mean contamination rates for WT and wMel.F were 0.88 and 0.25, respectively. (TIFF) [file pntd.0003894.s002.tiff]
