# Supplementary material for: Wolbachia Reduces the Transmission Potential of Dengue-Infected Aedes aegypti
Source: PLoS Negl Trop Dis. 2015 Jun 26;9(6):e0003894. doi: 10.1371/journal.pntd.0003894 (PMC4482661; doi:10.1371/journal.pntd.0003894)
Supplement: S1 Table — (DOCX) [file pntd.0003894.s003.docx]

Table S1. Virus integrity in sheep blood. Virus was titrated at 0 and 4 hours after DENV-3 was mixed with sheep blood.

| Dilution (fold) | Hour | Titer (Log_10_ TCID_50_/mL) |
| --- | --- | --- |
| - | 0 | 7.86 |
|  | 4 | 7.3 |
| 10 | 0 | 7.01 |
|  | 4 | 7.13 |
| 100 | 0 | 5.92 |
|  | 4 | 6.14 |
| 1000 | 0 | 5.09 |
|  | 4 | 4.65 |
| 10000 | 0 | 4.48 |
|  | 4 | 3.87 |
